# 임부의 스트레스, 우울 및 배우자와 가족의 지지가 모성 정체성에 미치는 영향

**DOI:** 10.4069/kjwhn.2020.03.17

**Published:** 2020-03-25

**Authors:** Hye-Jung Seo, Ju-Eun Song, Youngjin Lee, Jeong-Ah Ahn

**Affiliations:** 1 Ajou University Medical Center; College of Nursing, Ajou University, Suwon, Korea; 1 아주대학교병원, 아주대학교 간호대학; 2 College of Nursing·Research Institute of Nursing Science, Ajou University, Suwon, Korea; 2 아주대학교 간호대학·간호과학연구소소

**Keywords:** Pregnant women, Depression, Social support, Maternal health, 임부, 우울, 사회적 지지, 모성 건강

## Abstract

**Purpose:**

The objective of this study was to identify the factors influencing maternal identity in pregnant women.

**Methods:**

Using a descriptive research design, a cross-sectional survey was conducted. In total, 127 pregnant women were recruited from a tertiary hospital in Korea from January to April 2019. Measurements included maternal identity, stress, depression, spousal and familial support, and demographic and obstetric characteristics. Data were analyzed by descriptive statistics, the independent t-test, one-way ANOVA, Pearson correlation coefficients, and stepwise multiple regression using SPSS version 25.0.

**Results:**

The mean score for maternal identity was 131.15 out of 160, and the mean scores for stress, depression, and spousal and familial support were 14.59 (out of 40), 6.82 (out of 30), and 109.04 (out of 132), respectively. Stress (r=–.38, *p*<.001), depression (r=–.37, *p*<.001), and spousal and familial support (r=.37, *p*<.001) were significantly correlated with maternal identity. In multiple regression analysis, stress (β=–0.27, *p*=.005) and spousal and familial support (β=0.23, *p*=.014) were found to be significant factors influencing maternal identity in pregnant women (F=14.19, *p*<.001).

**Conclusion:**

It is necessary to develop effective strategies to mitigate stress and to encourage spousal and familial support in pregnant women. Such strategies could further enable pregnant women to enhance their maternal identity.

## Introduction

### 연구 필요성

모성 정체성은 임부가 어머니로서의 자신과 태아와의 상호성에 기초하여 모성 역할 행위를 인지하는 것으로[1], 이는 태아의 존재를 인식하는 순간부터 형성되어 출산 후에도 지속적으로 발달된다[2]. 여성에 있어 어머니가 된다는 것은 매우 중요한 발달기적 사건으로[3], 신체적 변화와 함께 정신적 및 사회적 변화를 초래하여, 임부가 임신 기간 동안 이러한 다양한 변화에 대해 적절히 적응하는 것이 매우 중요하다[3].

임부는 어머니로서의 새로운 역할과 수행에 대한 부담감, 또한 신체상 및 호르몬 변화 등으로 인하여 불안, 걱정, 두려움, 우울 등 부정적인 감정과 스트레스를 경험할 수 있다[4]. 그러나 임신으로 인한 임부의 다양한 심리적 변화는 정상 임부가 임신과정에서 겪는 변화이기도 하면서 동시에 임부의 심리·정신적 건강 문제로 발생할 수 있는 변화이기도 하므로, 이에 대해 특별한 관심을 가지거나 조기에 발견하기가 어려운 경향이 있다. 하지만 우울과 스트레스 등 임부의 부정적인 심리·정신적 경험은 임부가 임신과 출산에 대해 긍정적인 태도를 갖기 어렵게 하고, 어머니가 되는 기쁨보다 이를 상대적인 부담으로 느끼게 하여, 나아가 임부가 적절한 모성 정체성을 확립해 나가는 데 저해요소가 될 수 있다. 따라서 임부의 스트레스와 우울 등 심리·정신적 건강 상태에 관심을 가지고, 이들의 모성 정체성에 대한 영향 및 그 중요성을 파악하는 것 또한 필요하다 할 것이다.

또한 임부의 배우자와 가족의 지지는 임신 중 여성이 정체성과 자신의 삶의 의미를 수립하는 데 큰 영향을 미치며, 임신 기간 성공적으로 어머니가 되고자 하는 임부에게 원동력이 되어 준다[1]. 즉 배우자와 가족의 지지는 임부에게 임신과 출산이라는 사건을 긍정적으로 경험하게 하고, 임신과 관련된 스트레스와 어머니 됨에 대한 부담감 등을 감소시켜 심리적 안녕감을 제공하며[5], 나아가 모성 정체성을 발달시키는 데 큰 영향을 미칠 것으로 생각된다.

선행연구들을 살펴보면 모성 정체성 관련 요인으로 임부의 나이[6], 계획 임신 여부[7], 신체상[7], 자존감[8], 스트레스[8], 우울[6], 사회적 지지[1] 등이 보고된 바 있다. 그러나 정상 임부를 대상으로 우울과 스트레스 등의 심리적 요인과 배우자와 가족의 지지와 같은 사회적 요인이 통합적으로 모성 정체성에 미치는 영향을 파악한 연구는 찾아보기 어렵다. 특히 임신과 함께 정상적으로 발달되기 시작하는 여성의 모성 정체성은, 내적으로 부정적인 심리·정신적 요인은 감소시키고 외적으로 임신 전 과정 동안 긍정적 경험을 할 수 있도록 배우자와 가족의 지지를 촉진하여, 임신 초기부터 관심을 지니고 임신 전반에 걸쳐 적절한 형성과정을 거쳐야 한다. 이렇게 형성된 모성 정체성은 출산 후 여성 자신 뿐 아니라 태아와 가족에게 긍정적 영향을 미칠 것으로 기대된다.

따라서 본 연구는 임부의 일반적 및 임신 관련 특성과 함께 이들의 스트레스, 우울 및 배우자와 가족의 지지 정도를 확인하고, 궁극적으로 임부의 모성 정체성에 영향을 미치는 유의한 요인을 탐색하고자 하며, 이를 통해 향후 임부의 모성 정체성 증진과 나아가 모성 역할 적응을 돕기 위한 중재 프로그램 개발의 근거를 제공하고자 하였다.

### 연구 목적

본 연구는 임부의 스트레스, 우울 및 배우자와 가족의 지지가 모성 정체성에 미치는 영향을 확인하는 것으로 구체적인 연구 목적은 다음과 같다.

1) 임부의 스트레스, 우울, 배우자와 가족의 지지 및 모성 정체성의 정도를 확인한다.

2) 임부의 일반적 및 임신 관련 특성에 따른 모성 정체성의 차이를 검정한다.

3) 임부의 스트레스, 우울 및 배우자와 가족의 지지와 모성 정체성의 상관관계를 파악한다.

4) 임부의 모성 정체성에 유의한 영향을 미치는 요인을 탐색한다.

## Methods

Ethics statement: This study was approved by the Institutional Review Board of Ajou University Hosptal (IRB No. AJIRB-SBR-SUR-18-495). Informed consent was obtained from the subjects.

### 연구 설계

본 연구는 임부를 대상으로 스트레스, 우울, 배우자와 가족의 지지 및 모성 정체성의 정도를 확인하고, 모성 정체성에 영향을 미치는 요인을 탐색하기 위한 서술적 조사연구이다.

### 연구 대상자

본 연구의 대상자는 수원 아주대학교병원 산부인과에서 규칙적으로 산전 진찰을 받고 있는 임부를 대상으로 편의 표출하였다.

구체적 선정 기준은 1) 만 19세 이상의 기혼 여성, 2) 임신 15주 이상(임신 2, 3삼분기)의 초임부와 경임부, 3) 의사소통이 가능한 자, 4) 본 연구의 목적을 이해하고 참여에 동의한 자로 정하였으며, 제외 기준은 1) 임신 15주 미만(임신 1삼분기)의 임부, 2) 중증 기저질환이 있거나 관련 약물을 복용 중인 자(신체적 및 정신적 질환 포함), 3) 한국어를 읽고 이해하는 데 어려움이 있는 결혼 이민자 및 외국인 임부로 하였다. 연구대상자 수의 적절성을 평가하기 위하여, 유의수준 .05, 회귀분석에서 중등도 수준의 효과 크기(0.15), 검정력 .80, 예측 가능 변수 8개를 적용하여 G*power 3.1 프리웨어를 이용해 확인한 결과, 최소 표본의 수는 109명이었다. 선행연구[9]에서의 탈락률 약 25%를 고려하여 136명의 대상자에게 설문지를 배부하였고, 배부된 설문지 중 131부가 회수되었으며, 불충분한 응답의 설문지 4부를 제외하여 최종 127명 대상자의 자료를 연구의 분석에 이용하였다.

### 연구 도구

#### 모성 정체성

본 연구에서는 Kim과 Hong [10]이 개발한 임부의 모성 정체성 도구를 사용하였으며, 이는 모성 정체성의 행위적 요인 20문항과 정서적 요인 20문항의 총 40문항으로 구성되어 있다. 모든 문항은 Likert 4점 척도(‘아니다’ 1점, ‘매우 그렇다’ 4점)로 측정되고, 점수 가능 범위는 40–160점으로, 점수가 높을수록 예비 어머니로서 모성 정체성이 높은 상태임을 의미한다. 개발 당시 신뢰도는 Cronbach’s α=.93이었으며[9], 본 연구에서의 신뢰도는 Cronbach’s α=.92였다.

#### 스트레스

지각된 스트레스는 Lee 등[11]이 Perceived Stress Scale [12]을 한국어로 번안한 도구를 사용하여 측정하였다. 본 도구는 임부 및 산모를 대상으로 타당도가 검증된 바 있다[13]. 이는 대상자가 최근 1개월간 경험한 스트레스를 전반적으로 어떻게 지각하고 해석하는가를 묻는 것으로, 총 10문항으로 구성되어 있다. 모든 문항은 Likert 5점 척도(‘전혀 없었다’ 0점, ‘매우 자주 있었다’ 4점)로 측정되고, 점수 가능 범위는 0–40점으로, 점수가 높을수록 지각된 스트레스의 정도가 심한 것을 의미한다. Lee 등[11]의 연구에서 신뢰도는 Cronbach’s ⍺=.82였으며, 본 연구에서의 신뢰도는 Cronbach’s α=.85였다.

#### 우울

우울은 Han 등[14]이 Edinburgh Postnatal Depression Scale [15]을 한국어로 번안한 도구를 사용하여 측정하였다. 도구 개발 당시 본 도구는 산후우울증에 대한 선별검사 목적으로 개발되었으나, 이후 임신 중 우울에 대한 측정도구로서 국내외 연구에서 타당도를 확보하여 이용된 바 있다[16]. 본 도구는 총 10문항의 Likert 4점 척도(0–3점)로, 점수 가능 범위는 0–30점이며, 점수가 높을수록 우울이 심함을 의미한다. Han 등[14]의 연구에서 신뢰도는 Cronbach’s α=.85였으며, 본 연구에서의 신뢰도는 Cronbach’s α=.83이었다.

#### 배우자와 가족의 지지

배우자와 가족의 지지는 Prenatal Psychosocial Profile [17] 도구를 Kim [18]이 한국어로 번안한 도구를 사용하여 측정하였다. 본 도구는 총 22개 문항으로 배우자의 지지 영역 11문항과 가족 및 친지의 지지 영역 11문항으로 구성되어 있다. 모든 문항은 Likert 6점 척도(‘매우 불만족’ 1점, ‘매우 만족’ 6점)로 점수 가능 범위는 22–132점이며, 점수가 높을수록 배우자와 가족의 지지 정도가 높은 것을 의미한다. Kim [18]의 연구에서 신뢰도는 Cronbach’s α=.97이었으며, 본 연구에서의 신뢰도는 Cronbach’s α=.95였다.

#### 일반적 및 임신 관련 특성

대상자의 일반적 및 임신 관련 특성으로, 나이, 임신 주수, 산과력, 계획 임신 여부, 자연 임신 여부, 난임 시술 방법 및 횟수(해당되는 경우), 임신 관련 합병증 유무 등을 조사하였다.

### 자료 수집

본 연구는 2019년 1월부터 4월까지 자료수집을 진행하였다. 연구진행을 위하여 병원 간호부와 산부인과 주치의의 허락을 받았으며, 해당 병원 산부인과 외래를 방문한 임부 중 연구대상자 선정기준에 부합되는 자를 편의 표출하였다. 본 연구자와 함께 훈련된 간호사 1명이 연구의 목적과 방법, 연구대상자에 대한 윤리적 측면의 보호를 설명하고, 자발적 서면 동의를 취득한 후, 대상자에게 자가보고형 설문지를 배부하였다. 설문지 작성 시간은 약 20분 소요되었다.

### 자료 분석

수집된 자료는 IBM SPSS Statistics, ver. 25.0 프로그램( IBM Corp., Armonk, NY, USA)을 이용하여 다음과 같이 분석하였다.

1) 임부의 일반적 및 임신 관련 특성, 스트레스, 우울, 배우자와 가족의 지지 및 모성 정체성 정도는 빈도와 백분율, 평균과 표준편차로 분석하였다.

2) 임부의 일반적 및 임신 관련 특성에 따른 모성 정체성의 차이는 independent t-test, one-way ANOVA를 이용해 분석하였고, 사후검증으로 Scheffé test를 이용하여 분석하였다.

3) 임부의 스트레스, 우울 및 배우자와 가족의 지지와 모성 정체성의 상관관계는 Pearson’s correlation coefficient로 분석하였다.

4) 임부의 모성 정체성에 영향을 미치는 요인은 단계적 다중회귀분석(stepwise multiple regression)을 이용하여 분석하였다.

## Results

### 임부의 일반적 및 임신관련 특성

전체 대상자의 평균 연령은 만 33.20±4.03세였으며, 연령의 범위는 24–45세였다. 고령 임부의 기준인 만 35세를 기준으로 나누었을 때, 만 35세 미만은 80명(63.0%), 만 35세 이상은 47명(37.0%)이었다. 평균 임신 주수는 29.19±6.20주였으며, 임신 15–28주까지의 임신 2삼분기는 55명(43.3%), 임신 29주 이상의 3삼분기는 72명(56.7%)이었다. 분만력에 따라, 초산모는 76명(59.8%), 경산모는 51명(40.2%)이었고, 과거 유산 경험이 있는 경우는 38명(29.9%)이었는데, 이 중 습관성 유산인 경우는 5명이었다. 계획된 임신은 90명(70.9%)이었으며, 자연 임신이 된 경우는 88명(69.3%), 난임 시술을 받은 경우 39명(30.7%)으로 나타났다. 난임 시술자의 경우 시술 방법으로는, 체외수정 28명(71.8%), 인공수정 8명(20.5%), 배란유도는 3명(7.7%)이 받았다고 응답하였다. 난임 시술 횟수는 1회 시술한 경우 22명(66.6%), 2–4회 시술한 경우 9명(27.3%), 5회 이상의 경우 2명(6.1%)이었다(무응답 제외). 임신 관련 합병증을 진단받은 경우는 69명(54.3%)이었다(Table 1).

### 임부의 스트레스, 우울, 배우자와 가족의 지지 및 모성 정체성 정도

연구대상자의 스트레스 점수는 40점 만점 중 평균 14.59±5.56점이었다. 우울 점수는 30점 만점 중 평균 6.82±4.61점이었으며, 정상군(9점 이하)은 97명(76.4%), 우울군(10점 이상)은 30명(23.6%)으로 나타났다. 배우자와 가족의 지지 점수는 132점 만점 중 평균 109.04±17.19점이었으며, 하위 요인으로 배우자의 지지는 평균 56.16±9.47점(66점 만점), 가족 및 친지의 지지는 평균 52.88±10.38점(66점 만점)이었다.

연구대상자의 모성 정체성 점수는 160점 만점 중 평균 131.15±15.38점이었으며, 하위 요인으로 행위적 요인은 62.82±10.02점(80점 만점), 정서적 요인은 66.33±8.11점(80점 만점)이었다(Table 2).

### 임부의 일반적 및 임신 관련 특성에 따른 모성 정체성의 차이

연구대상자의 일반적 및 임신 관련 특성에 따른 모성 정체성은 습관성 유산 여부와 난임 시술 방법에서 통계적으로 유의한 차이를 보였다(Table 1).

습관성 유산 경험이 있었던 임부(114.40±25.74점)는 그렇지 않은 임부(131.84±14.57점)에 비해 모성 정체성이 유의하게 낮았으며(t=–2.54, *p*=.012), 난임 시술 방법에 따라 배란유도로 임신한 경우(115.33±7.77점)가 체외수정(135.96±11.56점)에 비해 모성 정체성이 유의하게 낮았다(χ2=6.16, *p*=.046).

### 임부의 스트레스, 우울, 배우자와 가족의 지지 및 모성 정체성의 상관관계

모성 정체성과 주요 연구 변수인 스트레스, 우울 및 배우자와 가족의 지지 간의 상관관계를 분석한 결과는 Table 3과 같다.

임부의 모성 정체성은 스트레스(r=–.38, *p*<.001) 및 우울(r=–.37, *p*<.001)과 유의한 음의 상관관계를 보였으며, 배우자와 가족의 지지(r=.37, *p*<.001)와는 유의한 양의 상관관계를 보였다.

### 임부의 모성 정체성에 영향을 미치는 요인

임부의 모성 정체성에 영향을 미치는 요인을 탐색하기 위해, 본 연구의 주요한 독립변수인 스트레스, 우울 및 배우자와 가족의 지지와 함께 단변량 분석에서 모성 정체성과의 관련성이 있는 것으로 설명된 특성인 습관성 유산 여부와 난임 시술 방법을 독립변수로 투입하여 다중회귀분석을 실시하였다. 독립변수에 대한 회귀분석의 가정을 검정하기 위해 다중공선성, 잔차, 특이값 등을 확인하였다. Durbin-Watson을 이용하여 오차의 자기상관을 검정한 결과 1.74로 자기상관의 문제는 없었고, 공차한계(tolerance)의 범위는 0.39–0.99로 1.0 이하, 0.1 이상으로 나타났으며, 분산팽창인자(variance inflation factor, VIF)는 기준치인 10보다 크지 않으므로 다중공선성의 문제는 없는 것으로 확인되었다. 잔차의 가정을 충족하기 위한 검정 결과 선형성, 오차항의 정규성, 등분산성의 가정을 만족하였으며, 영향력 분석을 위해 Cook’s distance 통계량을 이용해 분석한 결과 1.0을 초과하는 값이 없어 특이값이 없는 것으로 확인되었다. 따라서 회귀식의 가정이 모두 충족되어 본 회귀분석의 결과를 신뢰할 수 있었다.

다중회귀분석의 결과, 임부의 모성 정체성에 유의한 영향을 미치는 요인은 스트레스(β=–.27, *p*=.005)와 배우자와 가족의 지지(β=.23, *p*=.014) 순이었다. 즉, 스트레스가 낮을수록, 배우자와 가족의 지지가 높을수록 임부의 모성 정체성은 증가하는 것으로 나타났다. 본 회귀모형은 통계적으로 유의하였고(F=14.19, *p*<.001), 모형의 설명력은 17%였다(Table 4).

## Discussion

본 연구는 임부를 대상으로 모성 정체성 정도를 파악하고, 모성 정체성에 유의한 영향을 미치는 요인들을 확인하여, 향후 임부의 모성 정체성 증진과 나아가 모성 역할 적응을 돕기 위한 중재 프로그램 개발의 기초자료를 제공하고자 시행되었다.

본 연구결과, 임부의 모성 정체성은 평균 131.2점(160점 만점)으로 중등도 이상이었으며, 동일한 도구를 사용하여 임신성 당뇨 임부를 대상으로 측정한 선행연구[19]의 127.8점과 비슷하거나 약간 높은 수준이었다. 본 연구의 대상자는 1개 지역 대학병원에 내원하는 임부였으며 이들 중 난임 시술을 받은 자가 30.7%, 임신 관련 합병증을 진단받은 경우가 54.3%로 정상 임부가 함께 포함 조사되었음을 고려할 때, 특정 임신 합병증(임신성 당뇨)을 지닌 임부를 대상으로 모성 정체성을 측정한 선행연구와 비슷하거나 다소 상회하는 수준인 것으로 생각되었다.

연구대상자의 임신 관련 특성에 따른 모성 정체성의 차이를 분석한 결과, 모성 정체성은 습관성 유산 여부와 난임 시술 방법에 따라 통계적으로 유의한 차이를 보였다. 구체적으로, 3회 이상 유산 경험이 있는 습관성 유산 임부는 그렇지 않은 임부에 비해 모성 정체성 점수가 통계적으로 유의하게 낮았다. 이는 선행연구에서 습관성 유산을 진단받은 여성이 그렇지 않은 여성에 비해 스트레스가 높고, 긍정적 정서는 낮게 인지하며 부정적 정서를 높게 인지한다는 결과[20]와 맥락을 같이 한다고 볼 수 있다. 또한 유산을 경험한 임부를 대상으로 일상적인 산전 간호와 함께 심리적 적응을 높이는 프로그램을 적용하였을 때 모성 역할 자신감을 높였다는 선행연구[21]를 고려할 때, 습관성 유산 경험이 있는 임부가 스트레스를 낮추고 심리적 안정을 도모할 수 있도록 지지함으로써 궁극적으로 이들에게 모성 정체성 및 모성 역할 자신감을 증진시킬 수 있을 것으로 기대되며, 관련 중재의 개발 및 적용이 필요할 것으로 생각된다. 난임 시술 방법과 관련해서는, 체외수정을 시행받은 임부가 배란유도를 시행받은 임부보다 모성 정체성 점수가 통계적으로 유의하게 높았다. 난임 시술 방법과 관련하여 모성 정체성을 측정한 선행연구는 없어 직접 비교는 어려우나, 난임 시술을 받은 임부의 태아에 대한 애착이 우울과 유의한 음의 상관관계에 있고 우울이 태아 애착에 영향을 미치는 요인으로 나타난 선행연구[6]를 고려할 때, 특정 난임 시술 임부의 심리적 정서를 조기에 파악하고 이들의 심리적 안녕을 돕기 위한 적절한 관리 체계가 필요하다. 이는 난임 임부의 모성 정체성 및 나아가 태아 애착을 증진시킬 수 있을 것으로 여겨지며, 향후 난임 관련 임부를 대상으로 모성 정체성을 비롯한 사회심리적 관련 변수를 파악하고자 하는 지속적 연구의 시도가 필요할 것이다.

본 연구에서 임부의 모성 정체성은 스트레스 및 우울과 유의한 음의 상관관계를 보였고, 또한 임부의 배우자와 가족의 지지와는 유의한 양의 상관관계를 보였다. 이는 산후 6개월 이내 산모를 대상으로 한 선행연구[22]에서 우울이 높을수록 모성 정체성이 낮다고 보고한 결과나, 산후 4–6주 이내 산모를 대상으로 한 선행연구[23]에서 우울과 스트레스가 높을수록 모성 정체성이 낮고, 사회적 지지가 높을수록 모성 정체성이 높다고 밝힌 연구결과와 유사하다 할 수 있다. 기존의 모성 정체성 관련 연구들은 주로 산모를 대상으로 진행되어 왔으나, 본 연구에서 임부의 모성 정체성 역시 산모의 모성 정체성과 마찬가지로 스트레스, 우울 및 배우자와 가족의 지지와 유의한 상관관계가 있음을 확인했다는 것에 의의가 있다 하겠다. 모성 정체성은 여성이 임신과 동시에 모성으로서의 자신을 인식하고 적응하는 것으로, 긍정적 모성 정체성 형성을 위해서는 임부의 모성 정체성 관련 요인들을 파악하고 이들을 중재하기 위한 노력이 필요할 것이다. 선행연구에서 초임부를 대상으로 모성 정체성 발달 중재 프로그램을 제공하였을 때 출산 후에도 이들의 모성 정체성과 모성 역할 자신감이 지속적으로 높았던 것으로 보고된 바 있어[24], 여성의 임신기부터 긍정적 모성 정체성의 형성에 관심을 가지는 것은 매우 중요하다고 생각된다.

본 연구에서 임부의 모성 정체성에 영향을 미치는 유의한 변수들을 회귀분석을 통해 검증한 결과, 임부의 스트레스와 함께 배우자와 가족의 지지가 유의하게 영향을 미치는 것으로 나타났다. 즉, 스트레스가 낮을수록, 배우자와 가족의 지지가 높을수록 모성 정체성이 증가하였다. 선행연구에서, 임신성 당뇨 임부에게 스트레스 관리를 포함한 통합적 자가관리 프로그램을 제공하였을 때 이들의 모성 정체성 향상에 유의한 효과가 있음이 보고된 바 있다[25]. 이와 같이 산부인과 외래에서 기존에 통상적으로 시행하는 임부와 태아의 신체적 상태 확인과 관리에 더불어, 임부의 심리적 요인, 특히 스트레스 관리를 포함한 중재 프로그램의 시행이 필요할 것으로 생각되며, 이는 나아가 모성 정체성을 유의하게 증진시킬 수 있을 것으로 기대된다. 또한 본 연구에서 임부의 모성 정체성에 유의한 영향요인으로 밝힌 배우자와 가족 지지의 경우, 선행연구에서 사회적 지지가 태아 애착의 유의한 영향요인이 되며[26] 또한 모성 역할 적응의 유의한 영향요인[27]으로도 보고된 바 있어, 이들과 같은 맥락임을 알 수 있다. 미숙아의 어머니를 대상으로 사회적 지지 중재를 제공한 선행연구[28]에서는 이러한 중재가 모성 역할 자신감 향상에 유의한 효과가 있다고 보고한 바 있다. 배우자와 가족의 지지는 임부의 긍정적인 모성 정체성을 확립하기 위한 요인으로, 향후 임부의 친밀한 지지체계인 배우자를 비롯해 가족 및 친지의 적극적 지지를 격려하고 이를 활성화하기 위한 보다 구체적인 중재 전략의 마련이 필요할 것으로 생각한다.

반면, 본 연구에서 앞서 대상자의 임신 관련 특성에 따른 모성 정체성 차이 검증 시 유의한 변수였던 습관성 유산 여부 및 난임 시술 방법과, 상관관계 분석에서 유의한 변수였던 우울의 경우, 본 회귀분석을 통한 결과에서는 모성 정체성에 유의한 영향요인으로서 규명되지 못하였다. 그러나 과거 산모를 대상으로 우울이 모성 역할 적응에 유의한 영향요인으로 도출되지 않았음을 보고한 선행연구[23]와, 반대로 우울을 모성 역할 적응 및 모성 정체성의 주요한 영향요인으로 보고한 연구들[27]이 공존함을 고려할 때, 향후 모성 정체성의 다양한 관련 요인들을 고려하여 지속적 반복 연구를 통한 규명이 필요할 것이다.

본 연구는 한 대학병원 산부인과에 내원하는 임부를 대상으로 하여 모성 정체성과 영향요인을 파악하고자 한 횡단적 조사연구로서 연구결과의 일반화 및 변화의 양상을 파악하는 데에 제한점이 있으며, 또한 연구도구 사용에 있어 비록 임부를 대상으로 타당도가 검증되었다 할지라도 기존의 산후우울 측정도구 및 일반적 스트레스 측정도구를 사용한 점을 제한점으로 볼 수 있다. 그럼에도 불구하고 본 연구는 과거 산모를 대상으로 한 모성 정체성 관련 연구에서 나아가, 모성 정체성의 발달 초기가 될 임부의 심리·정신적 및 사회적 측면에서의 우울과 스트레스 및 배우자와 가족의 지지가 모성 정체성에 미치는 영향을 파악하고자 하였으며, 특히 스트레스와 배우자 및 가족의 지지가 정상 임부에서의 유의한 모성 정체성 영향요인임을 밝힘으로써, 향후 임부의 모성 정체성 증진 및 모성 역할 적응을 돕기 위한 간호중재 개발의 근거를 제공하였다는 점에서 의의가 있다.

## Conclusion

본 연구는 임부의 모성 정체성 정도를 파악하고 모성 정체성에 유의한 영향을 미치는 요인들을 확인하고자 시행되었다. 본 연구에서 임부의 모성 정체성 정도는 중등도 이상의 수준을 보였고, 스트레스, 우울 및 배우자와 가족의 지지와 유의한 상관관계를 보였으며, 특히 모성 정체성은 스트레스가 낮을수록, 배우자와 가족의 지지가 높을수록 유의하게 증가하는 것으로 나타났다. 본 연구결과를 토대로 임부의 모성 정체성을 증진시키기 위해 이들 변수를 고려한 간호중재 프로그램을 개발하고 적용하여, 향후 임부의 모성 정체성과 나아가 여성의 모성 역할 증진 및 태아 애착을 향상시킬 수 있을 것으로 기대된다.

본 연구결과를 토대로 다음과 같이 제언하고자 한다.

첫째, 본 연구는 1개 지역 대학병원에 내원하는 임부를 대상으로 조사하였으나, 추후 국내 다양한 지역의 대상자 수집을 통해 임신의 초기 과정부터 추후 출산 이후에 이르기까지 각 시기에 따른 모성 정체성의 영향요인을 파악하고자 하는 종단적 연구가 필요할 것이다. 둘째, 본 연구에서 차이 검증 및 상관관계 분석 시 유의하였으나 회귀분석에서는 제외되었던 습관성 유산 여부 및 난임 시술 방법과 우울 변수 등을 포함하여 다양한 관련 요인들을 고려한 모성 정체성 영향 요인에 대한 반복 연구가 필요할 것이다. 셋째, 본 연구에서 모성 정체성의 유의한 영향 요인으로 나타난 스트레스 및 배우자와 가족의 지지를 고려한 임부의 모성 정체성 증진 프로그램의 개발 및 효과 평가 연구가 필요할 것으로 생각된다.

## Figures and Tables

**Table 1.** Differences in maternal identity according to participants’ characteristics (N=127)

Variable	Categories	n (%)	Mean ± SD	t, χ^2^ or F	*p*
Age (year)	< 35	80 (63.0)	130.63 ± 16.91	–0.54	.59
	≥ 35	47 (37.0)	132.04 ± 12.47		
Gestational age	Second trimester	55 (43.3)	128.91 ± 18.18	–1.38	.172
	Third trimester	72 (56.7)	132.86 ± 12.71		
Parity	Primipara	76 (59.8)	132.78 ± 15.10	1.46	.146
	Multipara	51 (40.2)	128.73 ± 15.62		
History of abortion	Yes	38 (29.9)	130.63 ± 18.75	–0.25	.805
	No	89 (70.1)	131.37 ± 13.81		
Recurrent abortions	Yes	5 (3.9)	114.40 ± 25.74	–2.54	.012
	No	122 (96.1)	131.84 ± 14.57		
Planned pregnancy	Yes	90 (70.9)	132.46 ± 15.20	1.50	.136
	No	37 (29.1)	127.97 ± 15.56		
Pregnancy method	Spontaneous	88 (69.3)	130.41 ± 15.94	–0.81	.417
	Artificial insemination	39 (30.7)	132.82 ± 14.10		
Types of fertility treatment^†^	In vitro fertilization^a^	28 (71.8)	135.96 ± 11.56	6.16	.046
	Intrauterine insemination^b^	8 (20.5)	128.38 ± 18.77		c< a^‡^
	Ovulation induction^c^	3 (7.7)	115.33 ± 7.77		
Number of fertility treatments^†,§^	1	22 (66.6)	132.64 ± 15.44	1.38	.501
	2–4	9 (27.3)	136.67 ± 10.59		
	≥5	2 (6.1)	144.00 ± 1.41		
Obstetrical complications	Yes	69 (54.3)	131.78 ± 15.38	0.50	.615
	No	58 (45.7)	130.40 ± 15.48		

† Nonparametric test,

‡ Scheffé test,

§ Excluded no response.

**Table 2.** Participants’ stress, depression, spousal and familial support, and maternal identity (N=127)

Variable	Categories	n (%) or Mean ± SD	Range
Stress		14.59 ± 5.56	0–40
Depression	No (< 10)	97 (76.4)	
	Yes (≥ 10)	30 (23.6)	
	Total	6.82 ± 4.61	0–30
Spousal and familial support (total)	Support from spouse	56.16 ± 9.47	11–66
	Support from family and friends	52.88 ± 10.38	11–66
	Total	109.04 ± 17.19	22–132
Maternal identity (total)	Behavioral factor	62.82 ± 10.02	20–80
	Emotional factor	66.33 ± 8.11	20–80
	Total	131.15 ± 15.38	40–160

**Table 3.** Relationships among stress, depression, spousal and familial support, and maternal identity (N=127)

Variable	r (*p*)			
	Stress	Depression	Spousal and familial support	Maternal identity
Depression	.75 (< .001)	1		
Spousal and familial support	–.50 (< .001)	–.57 (< .001)	1	
Maternal identity	–.38 (< .001)	–.37 (< .001)	.37 (< .001)	1

**Table 4.** Factors influencing maternal identity in pregnant women (N=127)

Variable	B	SE	β	t	*p*
(Constant)	119.15	11.54		10.33	< .001
Stress	–0.73	0.26	–.27	–2.84	.005
Spousal and familial support	0.21	0.08	.23	2.49	.014
	R^2^=.19, Adjusted R^2^=.17, F (*p*)= 14.19 (<.001)				
